# The profile of the COvid-19 VACcination register SAFEty study in Sweden (CoVacSafe-SE)

**DOI:** 10.48101/ujms.v126.8136

**Published:** 2021-12-10

**Authors:** Rickard Ljung, Anders Sundström, Maria Grünewald, Charlotte Backman, Nils Feltelius, Rolf Gedeborg, Björn Zethelius

**Affiliations:** Swedish Medical Products Agency, Uppsala, Sweden

**Keywords:** COVID-19, SARS-CoV-2, pharmacovigilance, epidemiological, surveillance, monitoring, safety, efficacy, vaccine, signal detection

## Abstract

**Background:**

The coronavirus disease 2019 (COVID-19) vaccines have been rapidly implemented in national vaccination programs world-wide after accelerated approval processes. The large population exposure achieved in very short time requires systematic monitoring of safety. The Swedish Medical Products Agency has launched a project platform for epidemiological surveillance to detect and characterise suspected adverse effects of COVID-19 vaccines in Sweden.

**Methods:**

The platform includes all individuals 12 years or older in Sweden in 2021 and will be updated annually. Data, including vaccine and COVID-19 disease data, socioeconomic and demographic data, comorbidity, prescribed medicines and healthcare utilisation outcomes, are obtained from several national registers in collaboration with other Swedish Government agencies. Data from 2015 to 2019 are used as a historical comparison cohort unexposed to both the COVID-19 pandemic and to the COVID-19 vaccines.

**Results:**

The primary study cohort includes 8,305,978 adults 18 years and older permanently residing in Sweden on 31 December 2020. The historical control cohort includes 8,679,641 subjects. By 31 July 2021, around 50% of those 18 years and older and two-thirds of those 50 years and older were vaccinated with at least one dose, 90% of those 70 years or older had two doses.

**Conclusions:**

The nationwide register-based study cohort created by the Swedish Medical Products Agency with regular updates of individual level linkage of COVID-19 vaccination exposure data to other health data registers will facilitate both safety signal detection and evaluation and other pharmacoepidemiological studies.

## Introduction

The coronavirus disease 2019 (COVID-19) pandemic caused by severe acute respiratory syndrome coronavirus 2 (SARS-CoV-2) was declared as a Public Health Emergency of International Concern by the World Health Organization on 30 January 2020. Symptoms of COVID-19 are highly variable, ranging from none to life-threatening critical illness, and COVID-19 is related to more than 4.9 million deaths as of 21 October 2021 (1).

Several COVID-19 vaccines have demonstrated efficacy as high as 95% in preventing symptomatic COVID-19 infections. As of June 2021, two mRNA vaccines (Comirnaty^®^ and Spikevax^®^) and two viral vector vaccines (Vaxzevria^®^ and COVID-19 Vaccine Janssen) have been granted marketing authorisation in the European Union (EU). Several other vaccines are under evaluation for marketing approval.

Sweden implemented a phased vaccine distribution plan prioritising vaccination of those at highest risk of COVID-19 complications and those at high risk of exposure and transmission, e.g. healthcare workers, but at present (August 2021), all 16 years and older are eligible for vaccination. The Swedish Medical Products Agency participates in the assessment of marketing authorisation applications as well as the monitoring of safety of COVID-19 vaccines within the EU. The Marketing Authorization Holder has responsibility for monitoring safety post-marketing, and this is supervised by the European Medicines Agency.

In addition to the regular pharmacovigilance activities in place for all medicines, the Swedish Medical Products Agency has launched a project for epidemiological surveillance to detect and characterise suspected adverse effects of COVID-19 vaccines in Sweden. This study set-up is partly based on the valuable experience from the studies of narcolepsy undertaken following the nationwide vaccination campaign during the 2009–2010 H1N1 influenza pandemic (2–8). Narcolepsy as a serious adverse event of H1N1 influenza vaccine was first observed in Finland and Sweden and proved initially more difficult to detect in other countries, highlighting the importance of studies in national settings. Information on individual vaccination exposure was not directly available in a national vaccine register during the Pandemrix^®^ vaccination campaign in 2009–2010, leading to cumbersome efforts to retrieve this information from local authorities and regions (5). With the recent inclusion of COVID-19 vaccine data into the national vaccination register, individual level vaccination exposure data linked to other health data registers will facilitate extended possibilities for safety signal detection and evaluation, as well as other pharmaco-epidemiological studies. An in-depth safety surveillance is not only motivated by the concern for individual and population health but also to maintain the trust in national vaccination programs in general.

The overall aim of the present work is to describe the study design, major objectives and data sources, used for a nation-wide, population-based, pharmaco-epidemiological surveillance system.

## Material and methods

### Major objectives of COvid-19 VACcination register SAFEty study in Sweden (CoVacSafe-SE)

The major objectives of the CoVacSafe-SE are to study effectiveness and safety of vaccines for COVID-19 with a special focus on early detection and characterisation of suspected adverse events.

Specific aims are to:

- Study the occurrence of specific diagnoses, conditions and symptoms in vaccinated compared to non-vaccinated and historical cohorts.
- In in-depth analysis study the occurrence of suspected serious adverse events.
- Study mortality amongst vaccinated and non-vaccinated.
- Follow-up of vaccine effectiveness.
- Study the coverage of vaccination uptake.

### Study design and data collected

#### Study base

**Figure 1:**
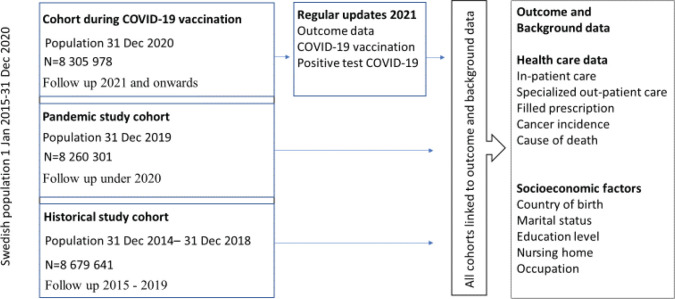
Flow chart of the COvid-19 VACcination register SAFEty study in Sweden (CoVacSafe-SE).

The study base comprises all individuals 12 years and older and alive and registered as permanently residing in Sweden on 31 December any year from 31 December 2014 to 31 December 2020. The study is comprised of three cohorts (Figure 1).

#### Study cohort during the COVID-19 vaccination in 2021

The cohort comprises all individuals 12 years and older permanently residing in Sweden, as registered on 31 December 2020 and consists of 8,305,978 adults (Table 1). This study cohort residing in Sweden during the COVID-19 vaccination in 2021 is the main cohort under study. For this cohort, information on COVID-19 diagnosis and exposure to COVID-19 vaccines is continuously collected, and the population is followed for subsequent outcomes of suspected adverse events. Occurrence of different diseases in vaccinated is compared to occurrence of diseases in non-vaccinated and to occurrence in the Swedish population in previous years (the historical cohort permanently residing in Sweden 2015–2019, and to the pandemic cohort permanently residing in Sweden in 2020 and exposed to the SARS-CoV-2 pandemic, see below). The vaccination campaign started in Sweden on 27 December 2020. Vaccination status as of 31 July 2021 is presented in Table 2, and positive COVID-19 status as of 1 January 2021 is presented in Table 1.

**Table 1:** Distribution of the adult population 18 years and older permanently residing in Sweden, as registered on 31 December 2020, included in the COvid-19 VAccination Register Safety Study in Sweden (COVARSS-SE), by 5-year age groups and sex, and the proportion with registered positive tests for COVID-19.

Age	Men		Women		Total
	Population	Positive COVID-19 2020	Population	Positive COVID-19 2020	
	*N* (%)	%	*N* (%)	%	*N* (%)
18–24	422,386 (5.09)	5.52	378,369 (4.56)	6.55	800,755 (9.64)
25–29	351,608 (4.23)	5.36	331,318 (3.99)	6.57	682,926 (8.22)
30–34	386,400 (4.65)	5.11	367,685 (4.43)	6.20	754,085 (9.08)
35–39	345,447 (4.16)	5.00	325,926 (3.92)	6.21	671,373 (8.08)
40–44	324,607 (3.91)	5.28	311,603 (3.75)	6.80	636,210 (7.66)
45–49	334,830 (4.03)	5.72	324,240 (3.90)	7.31	659,070 (7.93)
50–54	336,187 (4.05)	5.70	327,383 (3.94)	6.87	663,570 (7.99)
55–59	336,618 (4.05)	5.12	327,517 (3.94)	6.10	664,135 (8.00)
60–64	287,481 (3.46)	4.52	285,298 (3.43)	5.16	572,779 (6.90)
65–69	270,845 (3.26)	3.06	274,620 (3.31)	2.86	545,465 (6.57)
70–74	265,954 (3.20)	2.03	278,869 (3.36)	1.63	544,823 (6.56)
75–79	239,241 (2.88)	1.80	256,847 (3.09)	1.60	496,088 (5.97)
80–84	139,457 (1.68)	2.04	166,318 (2.00)	2.14	305,775 (3.68)
85–89	75,148 (0.90)	2.61	108,900 (1.31)	3.24	184,048 (2.22)
90–94	31,394 (0.38)	4.14	61,227 (0.74)	4.83	92,621 (1.12)
95+	8,230 (0.10)	5.01	24,025 (0.29)	6.77	32,255 (0.39)
Total	4,155,833 (50.03)	4.77	4,150,145 (49.97)	5.59	8,305,978 (100)

**Table 2:** Proportion of the Swedish population, on 31 December 2020, vaccinated with any COVID-19 vaccine, by age and sex. Vaccination status as of 31 July 2021.

Age	Men				Women			
	Unvaccinated	1 dose	2 doses	Total	Unvaccinated	1 dose	2 doses	Total
	*N* (%)	*N* (%)	*N* (%)	*N*	*N* (%)	*N* (%)	*N* (%)	*N* (%)
18–24	221,025 (52.33)	172,755 (40.90)	28,606 (6.77)	422,386	162,875 (43.05)	169,013 (44.67)	46,481 (12.28)	378,369
25–29	156,198 (44.42)	163,552 (46.52)	31,858 (9.06)	351,608	129,823 (39.18)	148,163 (44.72)	53,332 (16.10)	331,318
30–34	152,038 (39.35)	194,243 (50.27)	40,119 (10.38)	386,400	132,731 (36.10)	170,132 (46.27)	64,822 (17.63)	367,685
35–39	121,080 (35.05)	175,630 (50.84)	48,737 (14.11)	345,447	100,271 (30.76)	150,680 (46.23)	74,975(23.00)	325,926
40–44	92,277 (28.43)	150,459 (46.35)	81,871 (25.22)	324,607	73,545 (23.60)	134,518 (43.17)	103,540 (33.23)	311,603
45–49	73,081 (21.83)	123,945 (37.02)	137,804 (41.16)	334,830	59,057 (18.21)	106,483 (32.84)	158,700 (48.95)	324,240
50–54	55,891 (16.62)	60,949 (18.13)	219,347 (65.25)	336,187	48,692 (14.87)	50,628 (15.46)	228,063 (69.66)	327,383
55–59	47,980 (14.25)	38,329 (11.39)	250,309 (74.36)	336,618	41,737 (12.74)	31,411 (9.59)	254,369 (77.67)	327,517
60–64	32,366 (11.26)	13,951 (4.85)	241,164 (83.89)	287,481	29,350 (10.29)	12,473 (4.37)	243,476 (85.34)	285,299
65–69	25,311 (9.359	10,641 (3.93)	234,895 (86.73)	270,847	23,979 (8.73)	10,440 (3.80)	240,203 (87.47)	274,622
70–74	19,176 (7.21)	7,628 (2.87)	239,154 (89.92)	265,958	18,767 (6.73)	7,946 (2.85)	252,162 (90.42)	278,875
75–79	13,353 (5.58)	5,998 (2.51)	219,891 (91.91)	239,242	14,200 (5.53)	6,707 (2.61)	235,942 (91.86)	256,849
80–84	7,790 (5.59)	3,645 (2.61)	128,025 (91.80)	139,460	9,619 (5.78)	4,536 (2.73)	152,165 (91.49)	166,320
85–89	4,827 (6.42)	2,271 (3.02)	68,050 (90.55)	75,148	6,982 (6.41)	3,440 (3.16)	98,481 (90.43)	108,903
90–94	2,472 (7.87)	1,284 (4.09)	27,640 (88.04)	31,396	4,803 (7.84)	2,324 (3.80)	54,107 (88.36)	61,234
95+	886 (10.76)	411 (4.99)	6,935 (84.24)	8,232	2,580 (10.74)	1,200 (4.99)	20,249 (84.27)	24,029
Total	1,025,751	1,125,691	2,004,405	4,155,847	859,011	1,010,094	2,281,067	4,150,172

#### Historical study cohort 2015–2019 for comparison of disease occurrence

This historical cohort comprises all individuals 12 years and older permanently residing in Sweden, as registered on 31 December any year from 31 December 2014 to 31 December 2018 and consists of 8,679,641 adults. The cohort is followed up until 31 December 2019. This cohort is neither exposed to the COVID-19 pandemic in 2020 nor to COVID-19 vaccination in 2021.

#### Pandemic study cohort 2020 for comparison of disease occurrence

This cohort comprises all individuals 12 years and older permanently residing in Sweden, as registered and alive on 31 December 2019 and consists of 8,260,301 adults. The cohort is followed up until 31 December 2020. In this period, the COVID-19 was pandemic, and access to vaccine was negligible, with the first vaccinations starting 27 December 2020.

### Data collected

The cohort is continuously updated with information on exposure to COVID-19 vaccines, covariates and outcomes during 2021 (see below). All data collected are on individual level with a unique personal identifier, making it possible to link data from all registers to the same individual. For the health data registers below, it is mandatory by law for healthcare providers to report to the respective registers, and patients cannot decline such registration.

### Data on COVID-19 diagnosis and exposure to COVID-19 vaccines

#### The national vaccination register

The national vaccination register contains information on vaccination for COVID-19 since 1 January 2021. The information includes personal identifier and date of vaccination. The vaccine products are specified by a unique identifier based on a combination of brand name, substance, formulation, additionally batch number and dose number (for repeated doses). The completeness of registration within the child immunisation program is high, where 98.4% of children had at least one vaccination recorded; however, completeness for vaccination for COVID-19 has not been published (9). The register is held by the Public Health Agency of Sweden.

#### Register on surveillance of notifiable communicable diseases (SmiNet)

SmiNet contains information on notifiable diseases, which must be reported by the laboratories and the physician treating the patient, or performing an autopsy, in accordance with the Swedish Communicable Diseases Act. SmiNet includes personal identifier, date of disease occurrence, date of testing, date of positive test and diagnosis of notifiable infectious disease (10). The register is held by the Public Health Agency of Sweden.

### Data on comorbidity and health outcome

Previous hospitalisation and specialised outpatient care up to 5 years before cohort entry are collected. Also filled prescriptions 1 year before cohort entry are collected.

#### The Swedish patient register

The Swedish patient register comprises information on all in-hospital care and out-patient specialist care in Sweden. The information includes personal identifier, admission and discharge dates, whether hospitalisation was planned or acute, codes for discharge diagnoses and surgical procedures, whether discharged as deceased, to own private residence or other healthcare facilities, and type of department and hospital. It has nation-wide coverage regarding in-patient care since 1987 and specialised outpatient care since 2001 (11, 12). The register is a discharge register; hence, the admission is reported at time of discharge, also, there is no information on admission diagnoses. There is no information on primary care. During the study period, diagnoses were recorded according to the Swedish clinical modification of the 10th revision of the International Statistical Classification of Diseases and Related Health Problems (ICD-10-SE). *CoVacSafe-SE* includes all main and secondary diagnoses, and surgical procedures except those of the chapter O, P and Z in ICD-10-SE. These diagnoses are omitted, but the admission or visit is reported. The register is held by the National Board of Health and Welfare.

#### The Swedish cancer register

The Swedish Cancer Register was set up in 1958. Every clinician, pathologist and cytologist in Sweden must report a new primary malignancy. The Cancer Register includes primary malignancies and certain benign tumours and precancerous lesions (13, 14). In comparison to the National Patient Register, the proportion of non-reporting to the National Cancer Registry was estimated to be 3.7% in 1998 (13). The register is held by the National Board of Health and Welfare.

#### The Swedish prescribed drug register

The Swedish Prescribed Drug Register contains details of all the prescriptions dispensed in Sweden since 1 July 2005. It is updated monthly with around 100 million prescriptions dispensed each year (15, 16). It covers the entire Swedish population and includes information on unique personal identifier of the patient, age, sex, place of residence, and prescription information on substance, brand name, formulation and package dispensed amount, dosage (in free text) and unique expenditure and reimbursement, date of prescribing and dispensing, practice that has issued the prescription, and prescriber’s profession. Drugs are identified by a unique identifier for each specific combination of brand name, substance, formulation and package. Additionally, all drugs are classified according to the Anatomic Therapeutic Chemical Classification System (ATC) (17). The register only includes filled prescription, neither medicines sold over the counter nor medicines administered in in-patient care, out-patient care or primary care. *CoVacSafe-SE* includes all filled prescriptions on full seven-digit ATC codes. The register is held by the National Board of Health and Welfare.

#### The Swedish intensive care registry

The Swedish Intensive Care Registry (SIR) is a non-profit organisation established in August 2001 with the purpose to operate a national quality register of intensive care units (ICUs) in Sweden (www.icuregswe.org). In 2020, 81 out of all 83 ICUs reported data to SIR. The register contains information related to ICU care, including patient characteristics on admission to the ICU, reasons for admission and severity scores indicating baseline risk. Comprehensive information is also collected on procedures, complications, treatment strategy and monitoring of organ dysfunction (18).

#### The Swedish cause of death register

The Swedish cause of death register contains information on personal identifier, sex, date and country of birth, place of residence at time of death, date and underlying cause of death and contributing causes of death, place of death (hospital, nursing home or assistant living, private residence or other/unknown), autopsy type, whether the deceased had undergone a surgical procedure within 4 weeks prior and whether the death occurred abroad. Data are available for all deaths of Swedish residents since 1952, and it has a 99.2% completeness of causes of death; however, some causes of death are non-specific; hence, overall, 96% of deaths have a specific underlying cause of death recorded (19). The register is updated annually, but during the pandemic, all deceased with an underlying cause of death of COVID-19 have been continuously registered. Hence, cause of death due to COVID-19 is available with only some weeks delay, whereas other causes of death usually are finalised during late spring the year after. Date of death, without information on underlying cause of death, is available with a lag time of around 2 weeks. The register is held by the National Board of Health and Welfare.

### Data on socioeconomic and demographic factors

#### The Total Population Register

The Total Population Register contains information on personal identifier, date of birth, country of birth, place of residence, marital status, date of death and date of immigration and emigration (20). The register is held by Statistics Sweden.

#### The longitudinal integrated database for health insurance and labour market studies

The longitudinal integrated database for health insurance and labour market studies (LISA) contains information on a wide range of socioeconomic factors. Information on mother’s and father’s country of birth, educational level, socioeconomic position and occupation was retrieved from the register (21). The register is held by Statistics Sweden.

#### Register on persons in nursing homes

The register contains information on elderly and persons with physical, psychiatric or intellectual disabilities, given nursing care in nursing homes, their own homes or other institutions. For this study, the register is solely used to define elderly aged 65 years and older living in nursing homes (22). The register is held by the National Board of Health and Welfare.

### Definition and classification of some socioeconomic variables

Definition and classification of persons living in elderly nursing homes, marital status, country of birth (23), highest attained educational level and socioeconomic position are described in Appendix A. The distribution of marital status, country of birth and highest attained education level in the population is presented in Tables 3 and 4.

**Table 3:** Distribution of the adult population 18 years and older permanently residing in Sweden, as registered on 31 December 2020, included in the COvid-19 VACcination register SAFEty study in Sweden (CoVacSafe-SE), by marital status*, country of birth**, highest attained education level*** and by age groups for men.

Total, *N*	Men						Total
	18–34	35–49	50–64	65–74	75–84	85+	
	1,160,394	1,004,884	960,286	536,799	378,698	114,772	4,155,833
	*N* (%)	*N* (%)	*N* (%)	*N* (%)	*N* (%)	*N* (%)	*N* (%)
**Marital status** *							
Married	77,061 (6.64)	441,186 (43.90)	497,036 (51.76)	318,498 (59.33)	243,307 (64.25)	65,925 (57.44)	1,643,013 (39.5)
Unmarried	1,075,698 (92.70)	488,754 (48.64)	301,717 (31.42)	105,449 (19.64)	40,480 (10.69)	7,259 (6.32)	2,019,357 (48.6)
Divorced	7,561 (0.65)	73,786 (7.34)	155,709 (16.21)	97,849 (18.23)	62,698 (16.56)	13,123 (11.43)	410,726 (9.88)
Widowed	74 (0.01)	1,158 (0.12)	5,824 (0.61)	15,003 (2.79)	32,213 (8.51)	28,465 (24.80)	82,737 (31.99)
**Country of birth** **							
Sweden, both parents Sweden born	695,635 (59.95)	597,907 (59.50)	666,326 (69.39)	421,841 (78.58)	323,973 (85.55)	101,184 (88.16)	2,806,866 (67.54)
2nd generation, one parent Sweden born	94,744 (8.16)	66,506 (6.62)	66,858 (6.96)	26,329 (4.90)	8,089 (2.14)	970 (0.85)	263,496 (6.34)
2nd generation, both parents born outside Sweden	84,665 (7.30)	36,992 (3.68)	28,343 (2.95)	8,096 (1.51)	1,765 (0.47)	300 (0.26)	160,161 (3.85)
1st generation, born in Europe	85,770 (7.39)	120,045 (11.95)	97,569 (10.16)	50,313 (9.37)	35,455 (9.36)	10,169 (8.86)	399,321 (9.61)
1st generation, born outside Europe	199,580 (17.20)	183,434 (18.25)	101,190 (10.54)	30,220 (5.63)	9,416 (2.49)	2,149 (1.87)	525,989 (12.66)
**Highest attained education level** ***							**Total *N* = 2,995,439**
Missing	NA	55,695 (5.54)	18,169 (1.89)	6,100 (1.14)	5,184 (1.37)	1,949 (1.70)	87,097 (2.91)
Compulsory, up to 9 years of schooling	NA	113,218 (11.27)	133,238 (13.87)	129,547 (24.13)	125,879 (33.24)	49,794 (43.39)	551,676 (18.42)
Secondary education (vocational), 11 years	NA	131,255 (13.06)	365,401 (38.05)	157,383 (29.32)	84,919 (22.42)	21,746 (18.95)	760,704 (25.40)
Secondary education (academic), 12 years	NA	291,048 (28.96)	119,111 (12.40)	78,258 (14.58)	66,580 (17.58)	17,661 (15.39)	572,658 (19.12)
University education less than 3 years	NA	141,627 (14.09)	148,742 (15.49)	72,161 (13.44)	35,634 (9.41)	8,763 (7.64)	406,927 (13.58)
University education 3 years or longer	NA	272,041 (27.07)	175,625 (18.29)	93,350 (17.39)	60,502 (15.98)	14,859 (12.95)	616,377 (20.58)

* Information on those cohabitating without being legally married is not included.

** Categories briefly described, please see methods section for exact definition.

*** Information on education level in 2018 and is only presented for those 35 years or older in 2021. Total *N* = 2,995,439.

**Table 4:** Distribution of the adult population 18 years and older permanently residing in Sweden, as registered on 31 December 2020, included in the COvid-19 VACcination register SAFEty study in Sweden (CoVacSafe-SE), by marital status*, country of birth**, highest attained education level*** and by age groups for women.

Total *N*	Women						Total
	18–34	35–49	50–64	65–74	75–84	85+	
	1,077,372	961,769	940,198	553,489	423,165	194,152	4,150,145
	*N* (%)	*N* (%)	*N* (%)	*N* (%)	*N* (%)	*N* (%)	*N* (%)
Marital status*							
Married	130,579 (12.12)	476,087 (49.50)	487,721 (51.87)	306,585 (55.39)	205,480 (48.56)	42,760 (22.02)	1,649,212 (39.74)
Unmarried	931,136 (86.43)	378,800 (39.39)	240,704 (25.60)	81,664 (14.75)	31,730 (7.50)	9,396 (4.84)	1,673,430 (40.32)
Divorced	15,257 (1.42)	102,620 (10.67)	192,337 (20.46)	119,844 (21.65)	85,841 (20.29)	25,787 (13.28)	541,686 (13.05)
Widowed	400 (0.04)	4,262 (0.44)	19,436 (2.07)	45,396 (8.20)	100,114 (23.66)	116,209 (59.85)	285,817 (6.89)
Country of birth**							
Sweden, both parents Sweden born	657,138 (60.99)	567,629 (59.02)	643,349 (68.43)	427,663 (77.27)	360,230 (85.13)	167,450 (86.25)	2,823,459 (68.03)
2nd generation, one parent Sweden born	89,926 (8.35)	63,507 (6.60)	64,820 (6.89)	26,288 (4.75)	8,808 (2.08)	1,455 (0.75)	254,804 (6.14)
2nd generation, both parents born outside Sweden	80,451 (7.47)	34,521 (3.59)	27,579 (2.93)	8,185 (1.48)	1,932 (0.46)	458 (0.24)	153,126 (3.69)
1st generation, born in Europe	85,541 (7.94)	108,721 (11.30)	99,924 (10.63)	64,815 (11.71)	42,982 (10.16)	21,055 (10.84)	423,038 (10.19)
1st generation, born outside Europe	164,316 (15.25)	187,391 (19.48)	104,526 (11.12)	26,538 (4.79)	9,213(2.18)	3,734 (1.92)	495,718 (11.94)
Highest attained education level***							**Total *N* = 3,072,773**
Missing	NA	39,797 (4.14)	13,198 (1.40)	5,853 (1.06)	6,695 (1.58)	4,275 (2.20)	69,818 (2.27)
Compulsory, up to 9 years of schooling	NA	81,899 (8.52)	97,169 (10.33)	99,624 (18.00)	127,698 (30.18)	93,200 (48.00)	499,590 (16.26)
Secondary education (vocational), 11 years	NA	94,581 (9.83)	271,944 (28.92)	191,646 (34.63)	146,952 (34.73)	56,208 (28.95)	761,331 (24.78)
Secondary education (academic), 12 years	NA	216,834 (22.55)	161,693 (17.20)	60,776 (10.98)	28,747 (6.79)	7,875 (4.06)	475,925 (15.49)
University education less than 3 years	NA	132,464 (13.77)	154,797 (16.46)	86,235 (15.58)	46,635 (11.02)	13,448 (6.93)	433,579 (14.11)
University education 3 years or longer	NA	396,194 (41.19)	241,397 (25.68)	109,355 (19.76)	66,438 (15.70)	19,146 (9.86)	832,530 (27.09)

* Information on those cohabitating without being legally married is not included.

** Categories briefly described, please see methods section for exact definition.

*** Information on education level in 2018 and is only presented for those 35 years or older in 2021. Total *N* = 3,072,773.

### Database updates

The project will run at least until 31 December 2024. Annual updates on the Swedish population each year at 31 December are planned.

Additionally, registers on exposure, outcome and covariates are updated regularly:

- Information on COVID-19 diagnosis and exposure to COVID-19 vaccines from SmiNet and the national vaccination register is updated weekly.
- Information on date of death from the cause of death register is updated bi-weekly.
- Information on in-patient care and specialised outpatient care from the patient register is updated monthly. There is, however, up to 3 months delay in reporting. Hence, data retrieved in April will be complete for January. Preliminary analyses show that data retrieved in mid-May are complete up to and including care given in March. Hence, the average delay until complete reporting is around 6 weeks.
- Data on nursing care homes are updated monthly.
- Data on intensive care from the SIR are updated monthly.
- Data on filled prescriptions from the prescribed drug register are updated monthly, with average 2 weeks delay. Hence, data retrieved mid-May are complete for April.
- Data on emigration and immigration are updated once every third months.
- Data on cancer diagnoses are updated annually.
- Data on educational level, marital status and occupation are updated annually.

### Quality assurance and quality control

Most of the registers used are of high or very high quality, including very high or complete coverage, high completeness and high validity of registered information (11–16, 18–22). Together with the Swedish Medical Products Agency, the Public Health Agency of Sweden and the National Board of Health and Welfare continuously monitor the data used in this study. Registration of COVID-19 vaccines is new to the national vaccination registry; hence, initial lag in registration by healthcare vaccinators has been anticipated. The coverage and completeness of COVID-19 vaccination registration in the register is, however, high or very high (personal communication with the Public Health Agency of Sweden). For some suspected adverse events, it may be important to validate the recorded diagnoses, foremost using the patient register.

### Strength and limitations

The main strengths of the CoVacSafe-SE include the population-based cohort design with full nationwide coverage of the adult population, and complete follow-up. Other advantages include the availability and regular updates of complete data on positive COVID-19 PCR test and complete data on COVID-19 vaccination, and health outcomes and covariates from complete and valid nationwide Swedish registers.

The main limitations concern the nature of register data. Outcomes that are not severe enough to require healthcare utilisation in terms of hospitalisation or specialised out-patientcare will not be reliably captured. COVID-19 infections are only captured if a laboratory test is performed. The availability of testing has varied over time, and likelihood of being tested may also depend on factors such as severity of symptoms, age, socioeconomic factors and vaccination status. Hence, capture of mild disease is hampered by lacking testing capacity. There is no information on lifestyle factors, laboratory data or patient clinical parameters, apart from what is registered for those treated in ICU in the SIR register. Also, this study does not collect any data on pregnancies or conditions related to childbearing (chapter O in the ICD, and also not on Chapters P and Z). In the comparison of disease occurrence after vaccination in 2021 to previous disease occurrence, the historical cohort 2015–2019 is used as reference. The year 2020 is not included as during the pandemic, a large drop in non-acute healthcare utilisation was noticed. For some specific analyses, foremost those of complications to COVID-19, the cohort residing in 2020 under the pandemic has been used.

For some of the variables reflecting socioeconomic position, there is a possibility of misclassification. Marital status does not take into account those cohabitating without marriage. Thus, there is an underestimation of persons sharing household.

Not all persons have information on their own country of birth or that of their parents. Persons born in Sweden with one of their parents born in Sweden and the other where country of birth is unknown, or both of unknown country of birth, have been categorised as born in Sweden by Sweden born parents. For the older generation, this is probably true, as they may well have been born in Sweden by parents born in Sweden, but where that information is missing. However, for the younger population, this is probably an overestimation of parents born in Sweden.

The proportion of those with more than compulsory education level has increased over time in Sweden. Hence, analyses on education level must take birth year into account. For this study, where we at present have information on educational level in 2018, we only categorised those 35 years or older in 2021 as before that age not all have reached their highest attained education level.

The classification of socioeconomic groups is today a bit outdated as educational requirements for several occupations have changed in the last decades. However, it is used to measure socioeconomic position in the adult working age population in the 80s and 90s. For this study, it will only be used for those born in Sweden and 70 years or older in 2021, i.e. born 1951 or earlier, and hence 39 years or older in the 1990 Census.

## Discussion on the research potential and regulatory use

The current pandemic situation and introduction of new vaccines stimulated the need for new register-based regulatory tools for follow-up of their effectiveness and safety and facilitate non-interventional studies. In such studies, signals on possible side effects may be evaluated and support regulatory decision-making. Signals originating from the spontaneous reporting system can also more timely be further evaluated, compared to the situation during the 2009–2010 H1N1 influenza pandemic (4).

CoVacSafe-SE will foremost focus on studies of potential short- and long-term adverse events of special interest, vaccination coverage and effectiveness of vaccines. The longitudinal design with regular updates of both exposure and outcome will enable product-specific studies of all vaccines approved for use against COVID-19. Additionally, the linkage of health data to information on sociodemographic and socioeconomic factors enables studies on inequalities in health. If suspected adverse events arise where the evaluation of a potential causality requires additional data not currently available in CoVacSafe-SE, e.g. laboratory data or in-depth data on specific medical conditions, the CoVacSafe-SE can be linked to such information. Thus, CoVacSafe-SE will facilitate the study of many important research questions by using the large potential of population-based register research in settings with nationwide registers of high completeness and validity (20, 27, 28).

## Ethics considerations

CoVacSafe-SE contains sensitive data on individuals in the whole adult Swedish population. Hence, high security in the management of data is crucial. Sweden has implemented a phased distribution vaccination plan of the whole adult population. An in-depth safety surveillance, detecting and characterising potential side effects of vaccines, is not only motivated by the concern for individual and population health but also important in order to maintain the trust in national vaccination programs in general. Hence, a well-functioning systematic monitoring of the safety of vaccines is highly important. The sensitive data on individuals are pseudonymised with only a few persons within the project group having access to the data. All data are stored in a designated server area without connection to the internet, only used for analyses of sensitive data and with highly restricted and monitored access.

The study is approved by the Swedish Ethical Review Authority (2020-06859 and 2021-02186) and has conformed to the principles embodied in the Declaration of Helsinki. Consent to participate is not applicable as this is a register-based study (29, 30).

## Concluding remarks

The Swedish Medical Products Agency has launched a project platform with the dual purpose of epidemiological surveillance to detect and characterise suspected adverse events of COVID-19 vaccines in Sweden and scientific research. The nationwide study, COvid-19 VACcination register SAFEty study in Sweden (CoVacSafe-SE), using the newly implemented national register on individual level COVID-19 vaccination exposure data linked to other health data registers with high completeness and regular updates will facilitate extended possibilities for signal detection and evaluation, as well as other pharmaco-epidemiological studies.

## Data access and collaboration

Access to similar data requires permission. Apart from ethical approval from the Swedish Ethical Review Authority (29), researchers will also need approval from each register holder. The Swedish Medical Products Agency will consider proposals for research collaboration. The CoVacSafe-SE may only be used for studies related to vaccine safety, vaccine effectiveness, vaccine coverage, COVID-19 disease and for assessment of quality and validity of data included in the register. Enquiries can be submitted to the agency (registrator@lakemedelsverket.se with a copy to the corresponding author rickard.ljung@lakemedelsverket.se).

## Appendix A – Categorisation of some socioeconomic variables

### Living in elderly nursing home

Living in elderly homes was categorised using information from the register on persons in nursing homes and was defined as being 65 years or older and being recorded as living in nursing home the preceding month.

### Marital status

Marital status was retrieved from the total population register and categorised as 1) married, 2) divorced, 3) widowed and 4) never married. Same sex marital status (previously called registered partner) is included in the above. Persons cohabitating without being married are not captured in this categorisation. The categories 2) and 3) can be combined into a group ‘previously married’.

### Country of birth

Country of birth and mother’s and father’s country of birth were retrieved from the total population register and the longitudinal integrated database for health insurance and labour market studies, respectively. Country of birth was categorised into five mutually exclusive groups of region of birth by a combination of the person’s own country of birth and his or her parents’ country of birth, similar to the one used by Statistics Sweden (23): 1) Person born in Sweden and both parents born in Sweden or where their country of birth is unknown, 2) person born in Sweden by one parent born in Sweden, 3) person born in Sweden and both parents born outside Sweden, 4) person born in Europe and 5) person born outside Europe. Categories 2) and 3) can be seen as second-generation immigrants and categories 4) and 5) as first-generation immigrants.

### Highest attained education level

Highest attained education level was retrieved from the longitudinal integrated database for health insurance and labour market studies and categorised as: 1) compulsory, up to 9 years of schooling, 2) secondary education, usually vocational, 11 years, 3) secondary education, usually academic, 12–13 years, 4) less than 3 years of university education and 5) 3 or more years of university education.

### Socioeconomic position

Socioeconomic groups were categorised as defined by the socioeconomic classification by Statistics Sweden retrieved from the longitudinal integrated database for health insurance and labour market studies (24). This classification is based on the 1990 Census and assigns individuals a socioeconomic group foremost based on their occupation, work tasks and educational requirements for that occupation. Individuals were categorised into seven groups: 1) unskilled manual worker, 2) skilled manual worker, 3) lower non-manual, 4) intermediate non-manual, 5) higher non-manual, 6) self-employed and farmers and 7) unknown, missing.

### Healthcare workers

Healthcare workers were classified according to the Swedish Standard Classification of Occupations (SSYK) (25). Healthcare workers can be categorised and ordered by length of post-compulsory education: physician, registered nurse, licensed practical nurse and a group including nursing assistant, nurse aide and psychiatric aide. We also have information on occupations related to the healthcare sector, e.g. pharmacists, dentists and veterinarians.

According to the Swedish Medical Subject Headings (MeSH), the Swedish healthcare worker occupations groups above correspond to the following definitions (26): *Physician*: Individuals licensed to practice medicine. *Registered nurse*: Professionals qualified by graduation from an accredited school of nursing and by passage of a national licensing examination to practice nursing. They provide services to patients requiring assistance in recovering or maintaining their physical or mental health. *Licensed practical nurse*: Health personnel who do not hold professional degrees or credentials but have completed training and are licensed to provide routine patient care under the direction of registered nurses and physicians. *Nursing assistant, nurse aide and psychiatric aide*: Persons who assist in the routine care of patients in psychiatric or somatic care, usually under the supervision of the nursing department.
